# Organ distribution of transgene expression following intranasal mucosal delivery of recombinant replication-defective adenovirus gene transfer vector

**DOI:** 10.1186/1479-0556-6-5

**Published:** 2008-02-08

**Authors:** Daniela Damjanovic, Xizhong Zhang, Jingyu Mu, Maria Fe Medina, Zhou Xing

**Affiliations:** 1 Department of Pathology and Molecular Medicine, Centre for Gene Therapeutics, and M.G. DeGroote Institute for Infectious Disease Research, McMaster University, Hamilton, Ontario, L8N 3Z5 Canada

## Abstract

It is believed that respiratory mucosal immunization triggers more effective immune protection than parenteral immunization against respiratory infection caused by viruses and intracellular bacteria. Such understanding has led to the successful implementation of intranasal immunization in humans with a live cold-adapted flu virus vaccine. Furthermore there has been an interest in developing effective mucosal-deliverable genetic vaccines against other infectious diseases. However, there is a concern that intranasally delivered recombinant viral-based vaccines may disseminate to the CNS via the olfactory tissue. Initial experimental evidence suggests that intranasally delivered recombinant adenoviral gene transfer vector may transport to the olfactory bulb. However, there is a lack of quantitative studies to compare the relative amounts of transgene products in the respiratory tract, lung, olfactory bulb and brain after intranasal mucosal delivery of viral gene transfer vector. To address this issue, we have used fluorescence macroscopic imaging, luciferase quantification and PCR approaches to compare the relative distribution of transgene products or adenoviral gene sequences in the respiratory tract, lung, draining lymph nodes, olfactory bulb, brain and spleen. Intranasal mucosal delivery of replication-defective recombinant adenoviral vector results in gene transfer predominantly in the respiratory system including the lung while it does lead to a moderate level of gene transfer in the olfactory bulb. However, intranasal inoculation of adenoviral vector leads to little or no viral dissemination to the major region of the CNS, the brain. These experimental findings support the efficaciousness of intranasal adenoviral-mediated gene transfer for the purpose of mucosal immunization and suggest that it may not be of significant safety concern.

## Background

It is increasingly believed that respiratory mucosal immunization will trigger more effective immune activation and protection against respiratory infection caused by viruses and intracellular bacteria. Indeed, such understanding has led to the successful development and licensure of a live cold-adapted flu virus vaccine that is given intranasally to healthy humans of 5–49 years of age [1]. This flu vaccine was shown to be well-tolerated, safe and efficacious [1,2]. Recently, a replication-deficient recombinant adenovirus-based flu vaccine expressing hemaglutinin was evaluated in healthy human subjects following intranasal administration and was found to be safe and more effective than cutaneous patch immunization [3]. Mounting experimental evidence from us and others suggests that intranasal immunization with recombinant viral-vectored or adjuvanted protein vaccines is more effective than parenteral immunization against pulmonary tuberculosis [4-8]. Based on all of these findings, there has been a strong interest in promoting the development and ultimate application of additional vaccine candidates, particularly viral-vectored vaccines, for human intranasal immunization against respiratory pathogens.

Since the nose has been widely explored as a site of delivery of drugs to the cerebro-spinal fluid and the CNS [9], there is a concern that intranasally delivered recombinant viral-based vaccines may disseminate to the CNS via the olfactory tissue. In this regard, several recent studies using recombinant adenoviral gene transfer vectors expressing LacZ or placental alkaline phosphatase (P1AP) have localized the transgene products to the olfactory tissue following intranasal inoculation in rodents [10-12]. However, there has been a lack of quantitative studies to compare the relative amounts of transgene products in the respiratory tract, lung, olfactory bulb and brain after intranasal mucosal delivery of a viral gene transfer vector. In our current study, we have used fluorescence macroscopic imaging, luciferase quantification and PCR approaches to compare the relative distribution of transgene products or adenoviral gene sequences in the respiratory tract, lung, cervical draining lymph nodes, olfactory bulb, brain and spleen. The knowledge from our study helps address the important safety issue associated with genetic intranasal mucosal vaccination.

## Methods

### Experimental animals

Female BALB/c mice 6 to 10 weeks of age purchased from Harlan Laboratory (Indianapolis, IN, USA) were housed under SPF conditions. All experiments were conducted following the guidelines of the Animal Research Ethics Board of McMaster University.

### Intranasal inoculation of adenoviral gene transfer vectors

For whole organ GFP imaging analysis, a replication-defective recombinant human type 5 adenoviral vector expressing green fluorescent protein (AdGFP) was used at the dose of 5 × 10^7 ^pfu per mouse. For luciferase assay, a replication-defective recombinant human type 5 adenoviral vector expressing luciferase (AdLuc) was used also at the dose of 5 × 10^7 ^pfu per mouse. Transgene expression by both vectors is driven off of a murine CMV promoter which represents an optimal promoter for adenoviral vectors allowing high levels of transgene expression in various tissues [13,14]. An empty replication-defective recombinant human type 5 adenoviral vector (Addl) was used as a control at the same dose. All adenoviral vectors were amplified in 293 cells and purified according to the protocols that we previously described [15]. The level of replication competent adenovirus (RCA) contamination in these preparations is below 0.000001% as determined by infecting A549 cells for at least 17 days with serially diluted viruses. A549 cells are recommended as a commonly used cell line in the method of testing for RCA although an improved two-cell line bioassay has been developed for this purpose in cases where the transgene product may potentially interfere with the A549 system [16]. Before use, the adenoviral based vectors were diluted in PBS to a total volume of 25 μl/mouse. For intranasal inoculation, mice were first lightly anesthetized with isofluorane and the adenoviral preparation was delivered to a nostril drop-wise with a pipette as previously described [4,5]. For the assessment of whole organ GFP fluorescence imaging, one mouse was used for each time point per each vector treatment (including the control), and three independent experiments were performed to verify the results. For the luciferase assay, three mice were set up per time point/gene transfer vector treatment and one mouse was used as control for each time point/control vector. In a separate experiment, mice were infected with AdLuc and various organs were then harvested, fixed in 10% formalin, processed, and stained with hematoxylin and eosin (H&E) for histologic assessment. Two mice were used per time point and the mice were sacrificed at days 1, 3, 7 and 12 post-intranasal inoculation.

### Whole organ fluorescence imaging

At various times post-AdGFP i.n delivery, mice were sacrificed and the fresh tissues including trachea, lung, cervical lymph nodes, spleen, olfactory lobes and brain were harvested and immediately subjected to GFP fluorescence imaging. Imaging was carried out using the LEICA MZ16F fluorescence stereomicroscope with GFP2 filter and the processing software OpenLab 4.0.4 at proper zoom ranges. The exposure time for image taking was between 1 to 4 minutes varied with the intensity of fluorescence.

### Tissue preparation and luciferase assay

Tissues were wrapped in aluminum foil, snap-frozen in liquid nitrogen and stored at -70°C until homogenization. The frozen samples were thawed in 1× CCLR (Cell culture lysis reagent, Part No: E153A, Promega) in 15 ml polypropylene tubes (1 ml CCLR for brain, lungs and spleen and 400 μl for olfactory lobes, lymph nodes and trachea). Tissues were homogenized for 30 seconds with tubes in ice, and then spun for 10 min at 3,000 rpm at 4°C. Immediately, 20 μl of supernatants were transferred into a luminometer plate (Microtiter Plates, Part No 7571, Thermo) in duplicate for each sample. Luciferase and substrate reaction was carried out by using the Promega Luciferase assay system (Cat No E1500, Promega), which allows for less than 10^-20 ^moles of luciferase to be measured. Luciferase activity was quantified and expressed as relative luciferase units/per gram total tissue proteins on Microplate Luminometer TROPIX.

### PCR assay for detection of adenoviral gene sequences in the tissue

Four mice were i.n infected with AdLuc at the dose of 5 × 10^7 ^pfu per mouse and sacrificed three days post-infection. To extract tissue genomic DNA, whole organs were wrapped in aluminum foil, snap-frozen in liquid nitrogen and stored at -70°C. The organs were then thawed and homogenized for 30 seconds in TRIZOL^® ^Reagent (Cat No 15596-018, Invitrogen). Total tissue DNA was obtained according to the manufacturer's DNA Isolation Protocol. Polymerase Chain Reaction (PCR) amplification of a section of the adenoviral genome (primer annealing to the Ad5 genome in the vector: 5'-CGG AAC ACA TGT AAG CGA CG-3'; primer annealing to the mCMV promoter in the vector: 5'-GCT GGT CGC GCC TCT TAT AC-3'; expected size 720 bp) and the Glyceraldehyde 3-phosphate dehydrogenase (GAPDH) housekeeping gene as a control (forward primer: 5'-AAT GCA TCC TGC ACC ACC AAC TGC-3'; reverse primer: 5'-GGA GGC CAT GTA GGC CAT GAG GTC-3'; expected size 550 bp) was performed. For each tissue, 4 μl of DNA at ~0.025 μg/μl was subjected to 35 cycles of PCR. The PCR reactions were electrophoresed through a 1% agarose gel, stained with ethidium bromide, and visualized under UV light. All target and control PCRs were done in triplicate.

## Results and Discussion

### Intranasal instillation of replication-defective recombinant adenoviral vector

Intranasal (i.n) instillation has been widely used to derive transgene expression within the respiratory tract, but the delivery methods may vary depending on the purpose of gene transfer. In our current study, in order to adequately address the organ distribution issue related to intranasal mucosal vaccination, we have used the same method of i.n delivery and the dose of adenoviral vector that we have employed for the purpose of intranasal adenoviral-mediated vaccination against pulmonary tuberculosis [4,5]. More specifically as we described above, a relatively small volume of viral preparation was delivered i.n to mice that were lightly anesthetized and were in upright position. We have previously found that using a replication-defective adenoviral-vectored TB vaccine expressing M.tb Ag85A antigen represents an effective way of intranasal mucosal immunization against subsequent pulmonary M.tb challenge [4-6].

### Distribution of transgene product by organ fluorescence macroscopic imaging after intranasal adenoviral vector delivery

**Figure 1:**
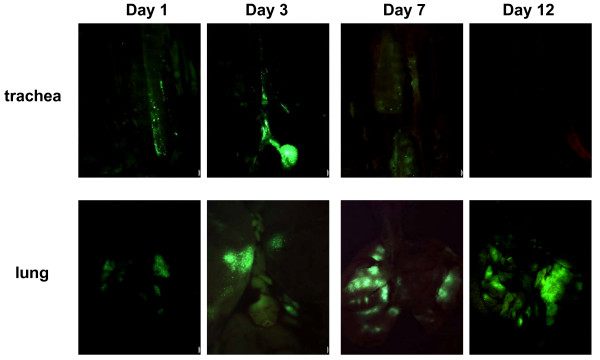
Fluorescence images of the trachea and lung following i.n delivery of AdGFP. After intranasal delivery of AdGFP, mice were sacrificed at days 1, 3, 7 and 12 (one mouse/time point) and freshly harvested organs were subject to fluoroscopic imaging. Magnifications (zoomrange): trachea ×20; lung ×7. The images are representative of three independent experiments.

To compare the relative distribution of transgene protein at various tissue sites following intranasal delivery of recombinant adenoviral gene transfer vector, we first used a recombinant replication-deficient adenoviral vector expressing green fluorescent protein (AdGFP). Use of this vector allowed us to conveniently assess the overall whole organ distribution of transgene protein by fluorescence macroscopy in freshly harvested tissues, without further tissue processing and manipulation. At days 1, 3, 7 and 12 following i.n delivery of a dose of 5 × 10^7 ^pfu AdGFP or a control Ad vector (Addl70-3), mice were sacrificed and their trachea, lung, olfactory bulb, brain, cervical draining lymph nodes and spleen were harvested and subjected to fluorescence macroscopy. As expected, no intense green fluorescence was detected at any time on organs of mice receiving control Ad vector (data not shown). However, following i.n delivery of AdGFP, there was patchy GFP expression on the interior surface of trachea at days 1 and 3 and subsequently declined (Fig. 1). By day 12, no GFP was seen in trachea. In comparison, by day 1 while GFP fluorescence was seen in the lung it intensified between days 3 and 7 (Fig. 1). By day 12 although the intensity decreased, GFP could still be seen in the lung. These overall kinetics of transgene expression in the lung are in agreement with our previous findings [17,18].

**Figure 2:**
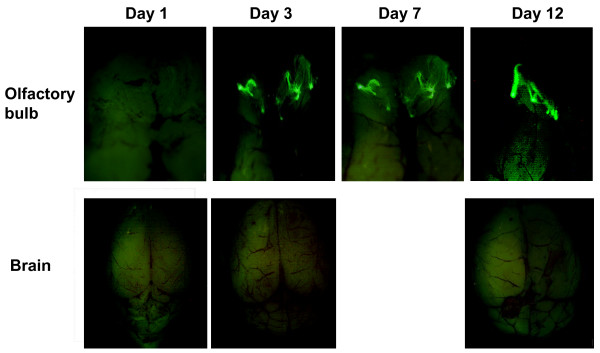
Fluorescence images of the olfactory bulb and brain following i.n delivery of AdGFP. After intranasal delivery of AdGFP, mice were sacrificed at days 1, 3, 7 and 12 (one mouse/time point) and freshly harvested organs were subject to fluoroscopic imaging. Magnifications (zoom range): olfactory bulb ×25; brain ×7. The images are representative of three independent experiments.

As there is evidence that in addition to infecting the epithelium of the respiratory system, intranasally delivered recombinant adenoviral vector may also infect the olfactory epithelium and neurons and subsequently the olfactory bulb via retrograde transport [10,11], we examined whether our i.n delivery method would also lead to viral gene transfer to the olfactory bulb of the CNS. Different from the respiratory tract, at day 1 we did not observe significant GFP in the olfactory region (Fig. 2). However, significant GFP was observed between days 3 and 12 (Fig. 2). These results suggest that in accord with studies by others [10], the intranasally delivered adenoviral gene transfer vector did get subsequently transported over to the olfactory bulb. The initial delay in transgene expression in the olfactory region, compared to relatively early expression in the trachea and lung (Fig. 1) may be due to the fact that the virus has to overcome the nasal/olfactory mucosal barrier and be transported via the olfactory nerve before it can reach the olfactory bulb.

As recombinant adenoviral gene transfer could reach the olfactory bulb as shown now by us and previously by others [10-12], it raises the question whether it may also reach the main part of the CNS, the brain. Upon examination of the brain excluding the olfactory lobes, however, we did not find any significant GFP (Fig. 2). This suggests that although i.n delivery leads to significant dissemination of adenoviral vector to the olfactory region, adenoviral vector unlikely affects other major parts of the CNS. Likewise, Lemiale and colleagues did not find any transgene product activities in the brain after i.n delivery of an adenoviral vector expressing placental alkaline phosphatase [10].

We found relatively faint GFP fluorescent patches/spots in the cervical draining lymph nodes, whereas we found no GFP at all in the spleen (data not shown). It was expected that small amounts of virus or virus-infected antigen presenting cells may migrate into the cervical lymph nodes which drain the nasal passage.

### Quantification of transgene product in various organs by luciferase assay after intranasal adenoviral vector delivery

**Figure 3:**
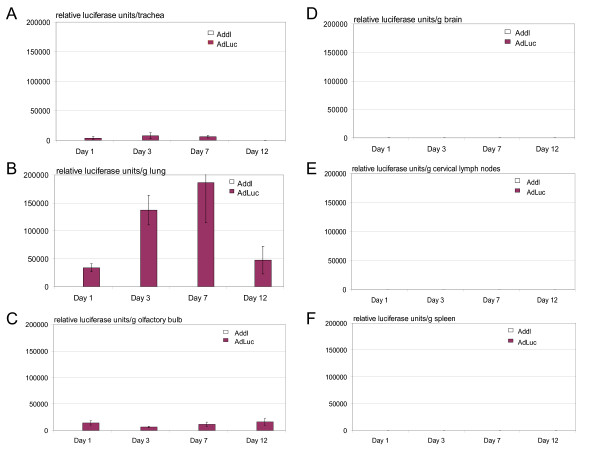
Quantification of luciferase activities in various organs following i.n delivery of AdLuc. After intranasal delivery of AdLuc, mice were sacrificed at days 1, 3, 7 and 12. The trachea (A), lung (B), olfactory bulb (C), brain (D), cervical lymph nodes (E) and spleen (F) were harvested and processed for the luciferase assay. Results are expressed as mean ± SEM from three mice/time point for AdLuc treatment. One mouse/time point was set up for naïve control measurement. The significance of differences in luciferase activities in the lung is as follows: it is significantly different between days 3/7 and day 1 or day 12 (p ≤ 0.01), but the difference between day 3 and day 7 is not significant (p = 0.1). There is no statistically significant difference between all time points in Fig. 3C.

In spite of the advantage of the fluorescence imaging technique, it cannot allow a quantitative assessment for comparison with regard to the extent of viral dissemination following i.n mucosal gene transfer. Furthermore, organ surface GFP imaging may miss viral infection that may have occurred in deep tissue. To this end, we used a recombinant replication-defective adenoviral gene transfer vector expressing luciferase (AdLuc) for i.n delivery and at various times after i.n, whole organs were homogenized and luciferase activities were quantified using the luciferase assay in the trachea, lung, olfactory bulb, brain, cervical draining lymph nodes and spleen. Consistent with GFP imaging, the trachea had moderate levels of luciferase activities following i.n gene transfer which could be detected from day 1 and declined to the baseline by day 12 (Fig. 3A). In comparison, of all of the organs examined, the lung produced the highest levels of luciferase activities which rose at day 1, peaked at days 3 and 7 and markedly decreased at day 12 (Fig. 3B). The levels of luciferase activities in the olfactory bulb, although also much lower than those in the lung, were in general higher than those in the trachea (Fig. 3C & Table 1), in basic agreement with fluorescence intensities detected in this tissue (Fig. 2). Upon comparison, the overall luciferase activities in the lung at peak times were 15–20 times that in the olfactory bulb (Table 1). However, compared to the lower trend of luciferase activities in other tissues by day 12, the level in the olfactory bulb at this time was still sustained. This could be due to a relative lack of inflammatory infiltrates in this tissue (see Table 2).

**Table 1:** Average values of luciferase activity in various tissues

	Trachea	Lung	Olfactory bulb	Brain	LN	Spleen
D1	3595	33728	14236	61	1	-23
D3	7718	136723	6794	29	4268	-4
D7	6046	186197	11677	49	83	4
D12	26	47428	16226	5	33	-3

The average luciferase units/gram tissue were determined from three mice/time point. The data are expressed as mean value of RLU/gram tissue proteins except the trachea (RLU/trachea) obtained by subtracting the background values of each set of control mouse tissues from the original measurement.

**Table 2:** Relative level of tissue inflammation after intranasal Ad inoculation

	Lung	Liver	Heart	Kidney	Brain
D1	-	-	-	-	-
D3	+	±	-	-	-
D7	+++	+	-	-	-
D12	+++	-	-	-	-

The grading of extent of tissue inflammation: "-" no inflammatory infiltrate seen; "+" or "± ", very mild inflammatory infiltration; "+++" significant inflammatory infiltration.

Consistent with the lack of GFP by fluorescence imaging, luciferase activities in the main part of the brain were negligible (Fig. 3D & Table 1), close to those in the negative control brains (Fig. 3F & Table 1). These thus further suggest the lack of any significant dissemination of intranasally delivered replication-defective recombinant adenoviral gene transfer vector to the large part of the CNS. This differs sharply from significant transgene expression detected in the olfactory region of the CNS (Fig. 2, Fig. 3C and Table 1), suggesting that such differential distribution of transgene product in the two different areas of the CNS is due to retrograde viral trafficking from the nasal mucosa, but not due to differential viral infectivity or promoter activities. By using a quantitative measure of transgene expression, our current study lends support to the conclusion drawn from other independent studies [10-12]. While the overall luciferase activities in the cervical lymph nodes were small, there was an unquestionable rise at day 3 (Fig. 3E) which was comparable to that in the trachea or olfactory bulb at the same time point (Table 1). This supports the draining property of these lymph nodes and suggests that these could be one of the primary immune activation sites following i.n mucosal vaccination [19]. The virus may directly disseminate via lymph and/or via infected antigen presenting cells to access the draining lymph nodes. With respect to the latter, we have recently observed in a separate study that fluorescently labeled dendritic cells, upon intranasal delivery, could subsequently be found in the cervical lymph nodes.

**Figure 4:**
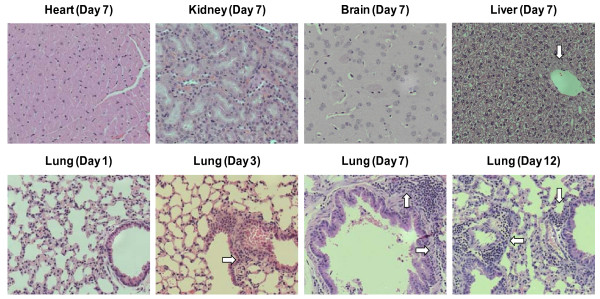
Histologic assessment of tissue inflammation after intranasal Ad inoculation. After intranasal delivery of AdLuc, mice were sacrificed at days 1, 3, 7 and 12 and the organs were fixed, processed, and stained with H&E. Open arrow: inflammatory infiltrates in the liver (day 7) and lung (days 1, 3, 7 and 12). These microhistographs are representative of two mice per time point. Magnification: ×20.

We have also assessed the level of inflammation in the lung, liver, heart, kidney and brain at days 1, 3, 7 and 12 following i.n AdLuc delivery. As shown in Table 2 and Figure 4, following i.n Ad inoculation, tissue inflammatory responses were seen primarily in the lung and to a much lesser extent in the liver (with very mild inflammatory infiltration in the perivascular area) while there was no inflammation in the heart, kidney or brain. The lack of inflammation in the heart and kidney may be due to the lack of viral dissemination. The lack of inflammation in the brain including olfactory bulb may be explained by several considerations: 1) the virus gets into the olfactory bulb via retrograde neurologic transfer but not through the brain-blood barrier; 2) the virus is replication-defective and does not replicate within brain cells, which does not cause the generation of sufficient chemotactic signals for leukocyte recruitment; and 3) the brain-blood barrier remains intact. Furthermore, lack of inflammation in the olfactory bulb region could explain the sustained levels of luciferase expression at this site, different from other tissue sites (Table 1).

### Distribution of adenovirus by PCR amplification after intranasal adenoviral vector delivery

**Figure 5:**
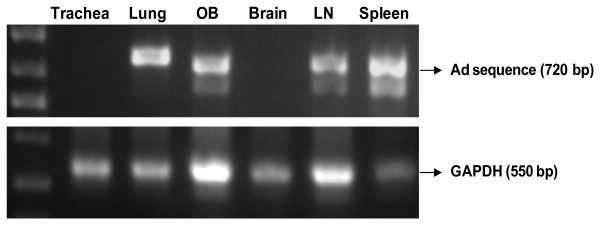
PCR amplification of an adenoviral genomic sequence after intranasal Ad inoculation. After intranasal delivery of AdLuc, mice were sacrificed at day 3 and the total DNA was isolated from organs. PCR amplification of adenoviral genomic sequences and the GAPDH control was performed using the isolated DNA as the template and primers outlined in the Methods. The PCR reactions were then electrophoresed through a 1% agarose gel, stained with ethidium bromide, and visualized under UV light. The data is representative of 4 mice. OB = olfactory bulb, LN = lymph node.

As transgene expression may vary depending on the relative promoter activities between tissues, it may underestimate the extent of adenoviral vector tissue dissemination. To this end, we further assessed viral dissemination by using PCR to detect adenoviral genomic sequences in various tissues following intranasal delivery. Mice were infected with AdLuc and at 3 days following infection, total DNA from various organs was analyzed using adenoviral genome-specific primers and PCR. Adenoviral gene sequence was not seen in the trachea (Fig. 5), which supports the patchy GFP expression and relatively very low luciferase activities. In comparison, a bright band was seen for the lung and olfactory bulb, in agreement with intense GFP and luciferase activities in these tissues (Fig. 5). Of note, the bright band observed for the spleen is in contrast to the lack of GFP and luciferase activity in this organ (Fig. 5). This observation suggests the dissemination of adenoviral vector to the spleen which is discordant with transgene expression, in agreement with the study by Johnson and colleagues, where intravenous administration of adenoviral vector resulted in a relatively high PCR adenoviral genomic signal in the spleen, but a low luciferase expression determined by optical imaging [20]. A possible contributing factor may be that the CMV promoter is less active in the resident immune cells in the spleen compared with lung cells for example [20]. Of importance, no adenoviral gene sequences were seen in the brain, which correlates with a lack of both GFP and luciferase activity, further supporting that the adenoviral vector does not disseminate to the brain (Fig. 5).

In conclusion, our results indicate that intranasal mucosal delivery of replication-defective recombinant adenoviral vector results in gene transfer predominantly in the respiratory system including the lung and transiently in the draining cervical lymph nodes, while it does lead to a moderate level of gene transfer in the olfactory bulb. However, intranasal inoculation of adenoviral vector leads to little or no viral dissemination to the major region of the CNS, the brain. These experimental findings support the efficaciousness of intranasal mucosal adenoviral-mediated vaccination. It is noteworthy that there have been no reports of brain-inflammation-related side effects after intranasal inoculation of Flumist – a live cold-adapted influenza virus vaccine or adenoviral-vectored vaccine in humans [1-3]. These observations together support the concept and feasibility of genetic-based intranasal vaccination in humans.

## Authors' contributions

DD and XZ performed the experiments, with support from JM and MM. ZX was the PI on this project. All authors read and approved the final manuscript.
